# Settings matter: a scoping review on parameters in robot-assisted gait therapy identifies the importance of reporting standards

**DOI:** 10.1186/s12984-022-01017-3

**Published:** 2022-04-22

**Authors:** Florian van Dellen, Rob Labruyère

**Affiliations:** 1 Sensory-Motor Systems Lab, Department of Health Sciences and Technology, ETH Zurich, Tannenstrasse 1, 8092 Zurich, Switzerland; 2 Swiss Children’s Rehab, University Children’s Hospital Zurich, Mühlebergstrasse 104, 8910 Affoltern am Albis, Switzerland; 3 Children’s Research Center, University Children’s Hospital of Zurich, University of Zurich, Steinwiesstrasse 75, 8032 Zurich, Switzerland

**Keywords:** Lokomat, Neurological rehabilitation, Gait speed, Body weight support, Robotic guidance, Public Reporting of Healthcare Data

## Abstract

**Background:**

Lokomat therapy for gait rehabilitation has become increasingly popular. Most evidence suggests that Lokomat therapy is equally effective as but not superior to standard therapy approaches. One reason might be that the Lokomat parameters to personalize therapy, such as *gait speed*, *body weight support* and *Guidance Force*, are not optimally used. However, there is little evidence available about the influence of Lokomat parameters on the effectiveness of the therapy. Nevertheless, an appropriate reporting of the applied therapy parameters is key to the successful clinical transfer of study results. The aim of this scoping review was therefore to evaluate how the currently available clinical studies report Lokomat parameter settings and map the current literature on Lokomat therapy parameters.

**Methods and results:**

A systematic literature search was performed in three databases: Pubmed, Scopus and Embase. All primary research articles performing therapy with the Lokomat in neurologic populations in English or German were included. The quality of reporting of all clinical studies was assessed with a framework developed for this particular purpose. We identified 208 studies investigating Lokomat therapy in patients with neurologic diseases. The reporting quality was generally poor. Less than a third of the studies indicate which parameter settings have been applied. The usability of the reporting for a clinical transfer of promising results is therefore limited.

**Conclusion:**

Although the currently available evidence on Lokomat parameters suggests that therapy parameters might have an influence on the effectiveness, there is currently not enough evidence available to provide detailed recommendations. Nevertheless, clinicians should pay close attention to the reported therapy parameters when translating research findings to their own clinical practice. To this end, we propose that the quality of reporting should be improved and we provide a reporting framework for authors as a quality control before submitting a Lokomat-related article.

**Supplementary Information:**

The online version contains supplementary material available at 10.1186/s12984-022-01017-3.

## Background

Over the last two decades, robot-assisted gait therapy (RAGT) has emerged as a frequently used technique in gait rehabilitation for patients with central neurologic gait disorders. Advantages, such as the possibility to achieve a high number of repetitions and reduced physical demands on the therapist, make RAGT an attractive option to clinicians. One of the most widely used robots is the Lokomat (Hocoma AG, Volketswil, Switzerland) which has been installed over 1000 times according to the company’s website [1]. Originally developed for people with spinal cord injuries [2], it has also been used for rehabilitation in numerous other conditions including stroke, Parkinson’s disease, multiple sclerosis and cerebral palsy [3–6]. Over the years, a significant number of clinical studies have been conducted to support the effectiveness of Lokomat therapy with scientific evidence. Most evidence was collected and analyzed in systematic reviews that found Lokomat therapy to be effective, but generally not superior to other forms of therapy like overground walking or manual treadmill therapy [3–7]. Looking more closely at the included studies, some showed an advantage over traditional training methods [8–17], but others failed to demonstrate this and urge therapists to remain cautious [18–27]. This heterogeneity of the results cannot easily be explained; for example, studies investigating people with the same diagnosis such as subacute stroke can be found distributed across that spectrum [14, 23].

The literature suggests that there might be various reasons for these mixed results. Several studies have investigated the influence of the patient population. For example, there is some evidence that more severely impaired patients with stroke might improve more than less affected patients [28]. Similar results have been found for children with cerebral palsy [29], although again this is controversial [30]. With respect to diagnosis, children with an acquired brain injury seem to benefit more than their peers with cerebral palsy [31]. Other attempts to find correlates between responsiveness and diagnostic factors in stroke [32] and spinal cord injury [33] have not been very successful.

Another important contribution to the mixed results might be differences in the therapy process itself. Modern rehabilitation research has established that goal-oriented therapy [34], a large amount of practice [35], and an active participation of the patient [36] are important contributors to a successful rehabilitation process. This suggests that the actual therapy content is important. The term RAGT describes the modality by which the therapy is administered, but it does not define the therapy content. In case of the Lokomat, therapy content is influenced by a range of parameters with which the therapy can be optimally adapted to the individual patient. The three most commonly adapted parameters available to all Lokomat users are the regulation of *gait speed*, the amount of unloading via a harness, the so-called *body weight support,* and a scaling factor for the forcefield that keeps the legs on the desired spatiotemporal trajectory, the so-called *Guidance Force* [2]. Additional features include virtual reality environments to increase patient motivation and activity via “gamification” of the therapy [37], the *FreeD module* [38] to facilitate a physiological weight shift, and a newer control mode named *Path Control* to allow temporal variability [39]. The availability of these additional features depends on the version of the Lokomat. There is some, albeit limited, evidence from clinical trials that the role of therapy parameters might be important [40, 41]. Kuo et al., recently could show in a retrospective study that the trajectory of therapy parameters during RAGT over time correlates with the improvement of walking function [42].

It can be concluded that the therapy content is a key aspect when interpreting the effectiveness of RAGT. Nevertheless, in the existing literature, it has not yet been sufficiently investigated how the therapy content, in terms of therapy parameters, influences therapy success. A potential reason for the limited evidence might be that, due to the heterogeneous patient groups trained, the ideal Lokomat parameters vary between patients. Several studies have highlighted the importance of the therapist in individualizing therapy [38, 43–45]. However, since there are currently no guidelines, each clinic or even therapist has developed their own opinion and preferences on how to adjust therapy parameters. Nevertheless, an appropriate reporting of the applied therapy parameters is key to the successful clinical transfer of study results.

The aim of this scoping review was therefore to evaluate how the currently available clinical studies report the applied Lokomat parameter settings and map the current literature on Lokomat therapy parameters. This should inform Lokomat practitioners about existing strategies and inform future research on tailoring RAGT.

## Methods

The protocol of this review was developed following the PRISMA extension for scoping reviews [46]. A completed checklist can be found in Additional file 1. The protocol of the scoping review was not published in advance. Three literature databases were searched for articles that investigated Lokomat therapy: PubMed, Embase and Scopus. Results were compared with the Hocoma literature database to identify potentially missed articles [47]. Search terms were designed to include at least one keyword from each of the following three categories: (1) study population (“patient”, “cerebral palsy”, etc.), (2) device (“Lokomat”, “robot” or “electromechanical”) and (3) activity (“walking”, “gait” or “Locomotion”). The exact search terms can be found in Additional file 2. The search results were retrieved on January 19th, 2021 and updated on February 22nd, 2022.

Titles, abstracts and full texts were screened independently by the two review authors to identify all original articles that met the following eligibility criteria: (1) The study population involved children, adolescents, or adults with a diagnosed neurological mobility impairment, (2) the study investigated Lokomat therapy. Reasons for exclusion were: (1) wrong devices (not Lokomat), (2) wrong study population (e.g. healthy subjects, orthopedic patients), (3) studies that focused only on assessments or technology, (4) wrong publication types (including reviews, editorials or letters) and (5) languages other than English or German. Conflicts were resolved by discussion until mutual agreement. The open source program Rayyan was used for the screening process [48].

The studies were divided into two groups: (1) *Clinical studies* that investigated the effectiveness of the therapy in multiple patients over multiple therapy sessions, (2) *other studies* that investigated Lokomat therapy, e.g. short-term effects within one therapy session. We assessed the quality of reporting for all *clinical studies* and used the articles from the group *other studies* as an additional source to provide an overview of the current evidence on the Lokomat parameters. Information about the therapy parameters, any rationale for their administration and any findings about these parameters were extracted from all included studies. In the scope of this study, therapy parameters included *body weight support*, *Guidance Force*, *Path Control* and *gait speed*. Although it is important to distinguish between *Guidance Force* and *Path Control* in the clinical context, in this text we group both parameters under the term *robotic assistance*.

### Reporting quality assessment framework

To assess the reporting quality of Lokomat therapy parameters, we developed a framework to evaluate the three most common parameters *gait speed*, *body weight support* and *robotic assistance* for all clinical studies. Four different categories were included to cover different aspects important for clinical transfer and the usability for a potential meta-analysis:

1. *Reference* Referencing a specific parameter illustrates that the therapists/scientists were aware of this parameter and readers can assume that certain considerations were made regarding this parameter.
2. *Strategy* This category refers to the mentioning of a strategy that guides the adjustment of a specific parameter. The reporting of the strategy is important as strategies might vary between studies. For example, the transfer of study results obtained by a therapy with a constant *gait speed* may not be comparable to results obtained by a therapy in a similar group of patients but with a progressively increasing *gait speed*. Mentioning a strategy therefore belongs to the minimum information needed by the reader to place study results within the context of their own work.
3. *Limits* The third category refers to setting boundaries for the therapy parameters. Often therapists do not use the full range of available therapy parameters. For example, therapists might never use a *body weight support* above 50%, nor reduce it below a minimum of 10%. A progressive reduction within these limits might mean something different than a progressive reduction from 100% *body weight support* to 50% *body weight support*. Therefore, this information complements the general strategy and helps to translate a therapy approach from a clinical study with promising results into the clinical setting.
4. *Actual settings* The intended strategy referred to in the first three categories might differ from the actually applied settings of parameters during therapy. For example, if authors intend to substantially decrease *Guidance Force*, but were not able to decrease it below 90%, this is a relevant information to judge whether the presented approach was successful. Moreover, knowledge about the actual settings is the main prerequisite for meta-analyses.

The complete framework with scoring rules can be found in Table 1. The points obtained for each individual category were added to a sum score per clinical study for each of the three parameters. The maximum number of points per parameter was 8. In addition, medians and interquartile ranges per parameter were calculated across all clinical studies to quantify the overall quality.

**Table 1:** Reporting quality assessment framework: For each study, the four questions are answered with one of the presented options. The corresponding points from all four categories are then added to a sum score per parameter

Categories	Questions	Answer options	Points	Example
Reference	Does the publication mention the parameter?	No	0	
		Yes	1	“Gait speed was individually set”
Strategy	Does the publication describe a strategy for the adaption of the parameter?	No	0	
		Yes	1	“Body weight support was gradually decreased as the patients improved”
Boundaries	Does the publication mention boundaries for the parameter settings?	No	0	
		Yes, one boundary	1	“Guidance force was lowered to a minimum of 40%”
		Yes, both boundaries	2	“Gait speed was initially set to 1 km/h and then increased to a maximum of 2.5 km/h”
Actual settings	Does the publication report the actually used parameter settings?	No	0	
		Yes, as a single group mean	1	“Patients trained with an average guidance force of 94% (standard deviation: 8%)”
		Yes, as group means over the course	2	“Initial body weight support was 44% (SD 6%) and could be lowered to 15% (SD 10%) over the course”
		Yes, as individual means	2	The study includes a table with the averaged settings per subject over all trainings
		Yes, as individual means over the course	3	The study includes a table with an average setting per subject for each the intial training and the final training
		Yes, as individual means over the course and within single therapies	4	The study includes Lokomat output files

### Levels of reporting quality

To provide guidance for authors of future Lokomat publications, we introduced a traffic light system for the reporting of Lokomat parameters. Studies with 0–2 points were categorized to have a poor reporting quality. These studies do not allow a transfer of a therapy approach to clinical practice. Studies with 3–6 points were categorized to have a limited reporting quality. These studies allow some transfer of strategies but are not eligible for meta-analyses. Finally, studies with the highest scores, 7–8, were categorized to have a sufficient reporting quality and are expected to be eligible for full clinical transfer and suitable for meta-analyses.

## Results

**Fig. 1:**
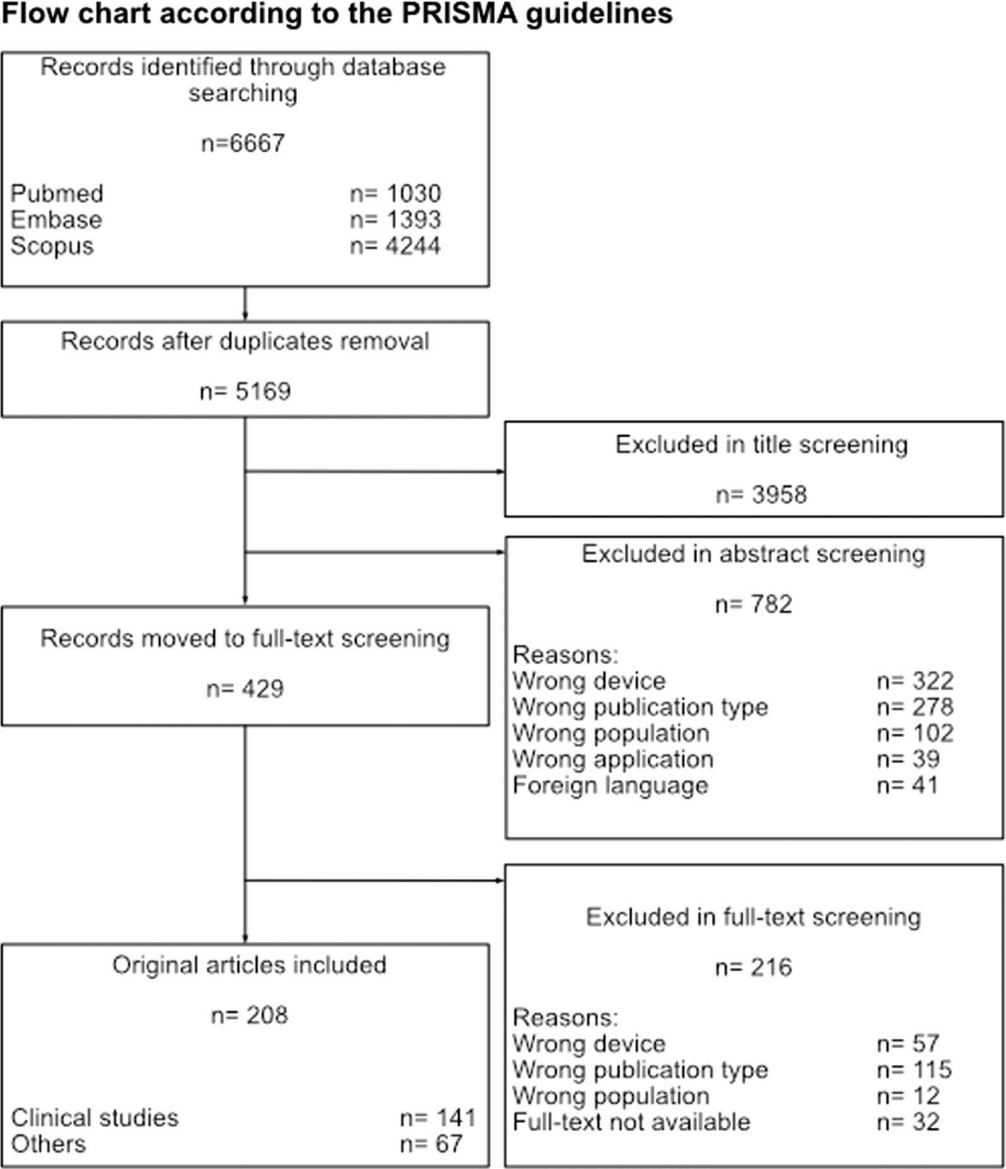
Flowchart presenting the workflow of the study identification and selection

The complete overview of the screening and selection procedure according to the PRISMA guidelines [49] is shown in Fig. 1.

The reporting of the *gait speed* in clinical studies was rated with a median of 3 and an interquartile range (IQR) of 2, the reporting of the *body weight support* with 3 (IQR: 3) and the reporting of the *robotic assistance* with 1 (IQR: 3.5). All individual sum scores by study and parameter can be found in Additional file 3.

**Fig. 2:**
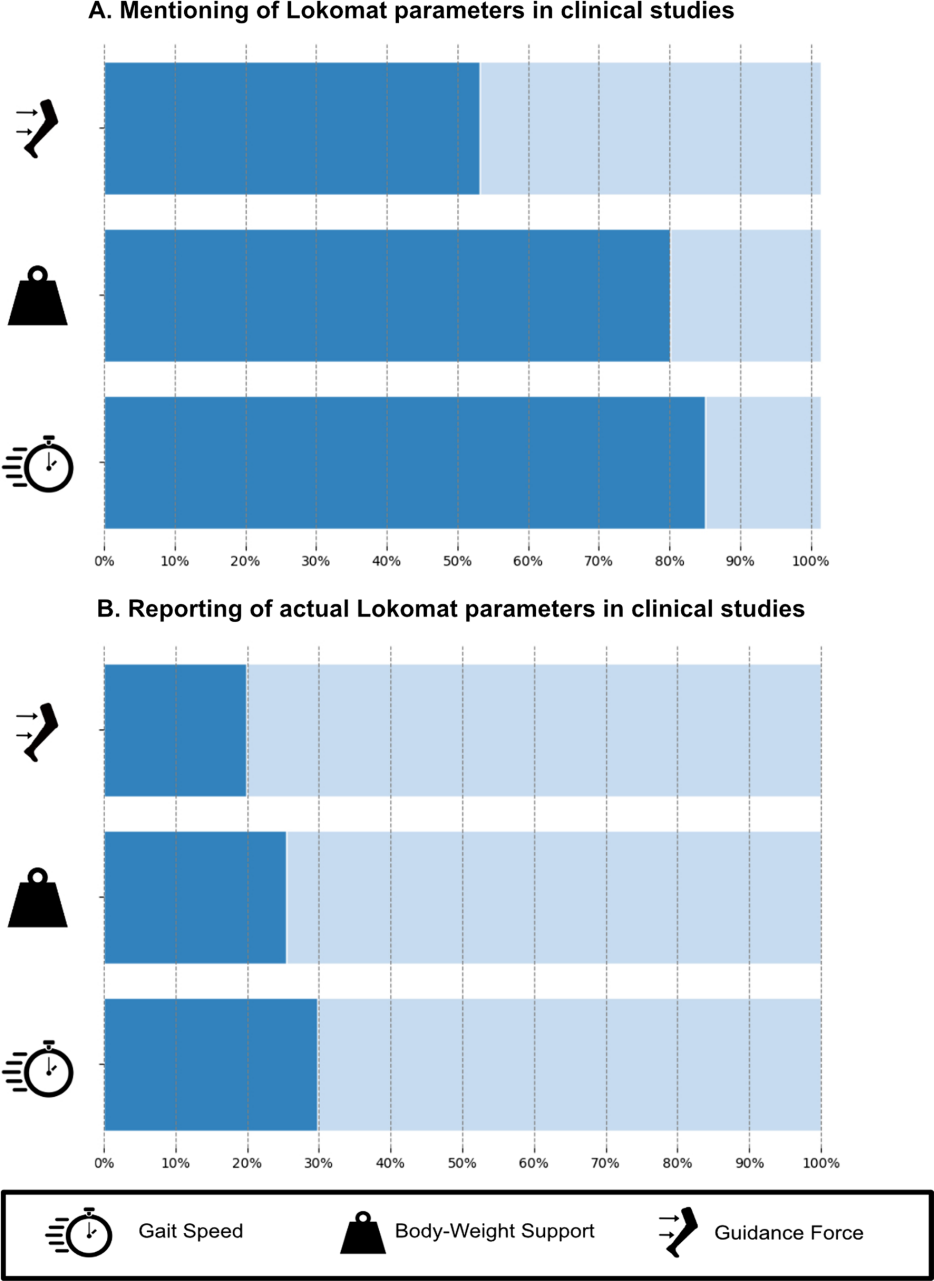
The dark portion in **A** illustrates the number of studies that mentioned the given Lokomat parameter. This corresponds to all studies that received one point in category *Reference* within the reporting framework. In **B**, the dark portion refers to all studies that reported the actual Lokomat parameter setting in some form. This corresponds to at least one or more points in category *Actual Settings* within the framework

Within the first category, Reference, 83% of the clinical studies mentioned the *gait speed* and thus received 1 point, followed by 79% that mentioned the *body weight support* and 51% that mentioned the *robotic assistance* (Fig. 2A). In terms of the subdivisions of *robotic assistance,* a single clinical study mentioned the application of *Path Control Guidance Force*.

**Fig. 3:**
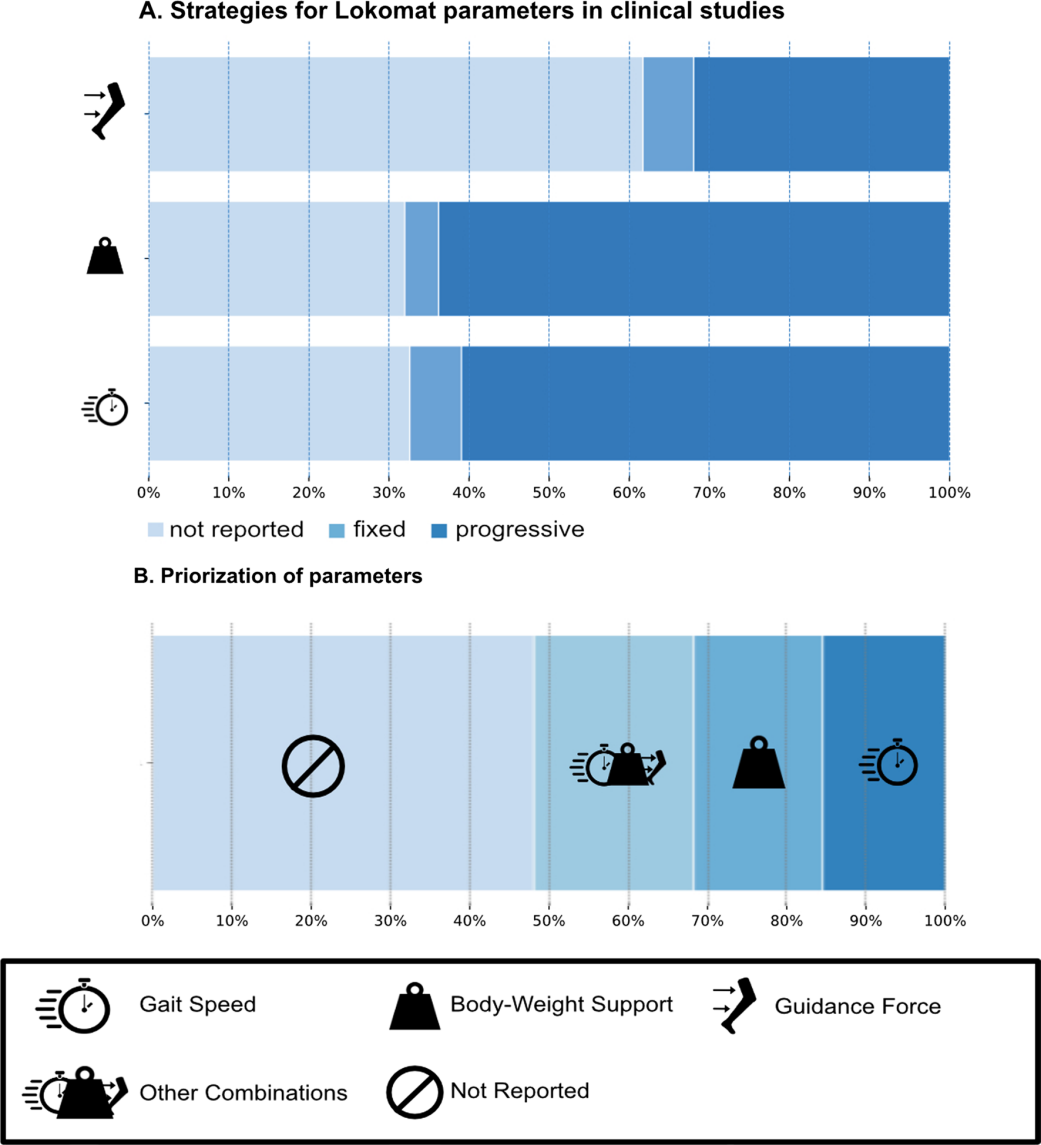
**A** The strategies employed for the Lokomat parameter setting reported by the clinical studies. **B** Illustrates the parameter that is adjusted first and prioritized over the other therapy parameters

Within the second category, Strategy, 69% reported a strategy for the *gait speed* and *body weight support* and thus received 1 point. Forty-one percent reported a strategy for the parameter *robotic assistance*. In detail, most of the studies progressively increased *gait speed* and decreased *bodyweight support* (Fig. 3A). Six percent of the clinical studies reported a fixed *gait speed*, around 4.2% of the studies reported a fixed *body weight support*. A progressive reduction in *Guidance Force* was less common, but was still reported in 32% of the studies whereas 6.3% reported a fixed *Guidance Force*.

Around 50% of the clinical studies reported in some form, which parameter they prioritized to personalize or progress the therapy. *Body weight support* and *gait speed* were cited as the first or most important parameter in 15% and 14%, respectively, of the studies (Fig. 3B). About 18% reported simultaneous adaptions or combinations of more than one parameter and only one single study reported Guidance Force as the most important factor. Approximately half of the studies did not report any kind of prioritization of parameters.

For the third category, Limits, 38% of all clinical studies reported one limit for the *gait speed* and thus received 1 point, and 16% of all clinical studies reported both lower and upper limits and received the maximum of 2 points. For the *body weight support*, 30% of the clinical studies reported one limit and 30% both limits. Finally, for the *Guidance Force*, 28% reported one limit and 7% both limits.

Less than one-third of all clinical studies reported information about the Actual settings used (Fig. 2B). Most commonly, an actual *gait speed* was reported in 28% of the cases, followed by *body weight support* (18%), and *Guidance Force* (17%). These fractions correspond to all studies that received at least 1 point in the category *actual settings*.

## Discussion

The present scoping review is, to the best of our knowledge, the first article summarizing the reporting of and evidence on the choice of Lokomat parameters. The results clearly illustrate that therapy parameters play a minor role in most of the currently published clinical studies. While there are numerous systematic reviews summarizing the clinical evidence for RAGT for different pathologies, the majority has not paid much attention to the actual therapy content of RAGT. Figure 2 illustrates that many authors appear to consider the parameters in some form, but only a small fraction provides detailed information about their actual settings in therapy. The more detailed the reporting according to our framework needs to be, the lower the proportion of studies that meets the requirements for points within these categories. This might seem logical, as a more detailed reporting requires more effort by the authors, but this decrease in reporting quality might also indicate a discrepancy between the importance therapists attribute to the parameters and the attention that they receive in the literature. While the high number of studies that consider at least some form of adjustment indicates that some importance is attributed to Lokomat parameters, the low quality of the reporting illustrates that the parameters are largely neglected in the scientific debate.

Despite the poor reporting quality within our framework, some information on the therapy parameters is available and we found that various strategies for their adjustment exist. While these different strategies might potentially be due to specific rehabilitation goals or different patient populations, it is currently not possible to underline most of these strategies with evidence and further clarifying studies are necessary. This also applies to differences and similarities across diagnoses.

The evidence currently available for therapy parameters comes predominantly from research-oriented studies that investigated reactions of patient groups during a single therapy session. Strategies and key findings on Lokomat parameters are summarized in the following sections.

### Gait speed

While most of the studies tend to increase the *gait speed* as the patients improve [10, 14, 20, 21, 24, 50–57], others even reduce the treadmill speed [41, 58–61]. Little research has been conducted on the influence of *gait speed*. However, research suggests that an increase in *gait speed* also increases the heart rate and thus the intensity of the therapy [62]. Along the same lines, research on patients with stroke and cerebral palsy suggests that an increase in *gait speed* increases the muscle activation [63, 64]. Nevertheless, there is some evidence that supports a reduction in walking speed with the purpose of activating supraspinal centers [41, 58, 60, 61].

### Body weight support

Variable strategies do also exist for the *body weight support.* While in the majority of the studies, the support never exceeded 50% of the body weight [8–10, 14, 15, 18, 19, 21, 22, 24–27, 32, 41, 50–52, 54, 56, 58, 65–95], other studies never decreased the support below 50% [96–102]. Current evidence on the *body weight support* suggests that a decrease in the *body weight support* can increase the metabolic costs [103] and can elicit higher heart rates [62]. This is related to the fact that a reduction in *body weight support* elicits a higher muscle activation [63, 64, 104, 105]. However, the muscle synergies were found to be robust across different levels of *body weight support* [105]*.* Besides these direct effects, there is some evidence that the *body weight support* interacts with the effects of other therapy parameters. For example, a high *body weight support* attenuated the effects of *gait speed* and *Guidance Force,* suggesting that load bearing is crucial for an adequate patient activity [63].

### Robotic assistance

While some therapists are in favor of minimizing the interactions between the robot and the patients [106] and aim for a low Guidance Force, others proposed to perform resistance training with the Lokomat [57]. The current evidence stems mostly from research oriented studies which generally take a look at short term effects but not the effectiveness of the therapy. König et al. found that a reduction of the *Guidance Force* did not significantly increase heart rate [62]. However, a reduction of the *Guidance Force* can increase the muscle activation [63, 64, 104]. Cherni et al. found a non-trivial influence of *Guidance Force* on muscle activations, partially attenuated previous results [105]. A pilot study could also show that during learning a trajectory tracking task in the Lokomat with a low *Guidance Force*, muscle activation could be increased and tracking errors could be reduced [106]. For *Path Control*, research suggests that its application normalizes the muscle activation patterns [38, 107].

Although these findings emphasize that therapy parameters alter the physiologic responses during the therapy and might be important to consider, they do not allow conclusions to be drawn about the effectiveness of specific adjustments of therapy parameters on therapy success. Further research is needed to advance the field and enable therapists to choose the best possible therapy parameter setting for their patients. The simplest form to gather additional evidence would be clinical studies where single parameters are being manipulated as it has been done in a very limited number of studies. For example, Park et al. [40], albeit using a different device, were able to show that reducing robotic assistance could be beneficial for the rehabilitation outcome. Similarly, Rodrigues et al. could show that the *gait speed* could influence the success of the training program [41]. However, simple manipulations of a single parameter might not be sufficient as complex interactions exist between the different therapy parameters [63]. Designing clinical studies that investigate combined effects of parameters would require an tremendous effort that hardly any clinic can afford.

**Fig. 4:**
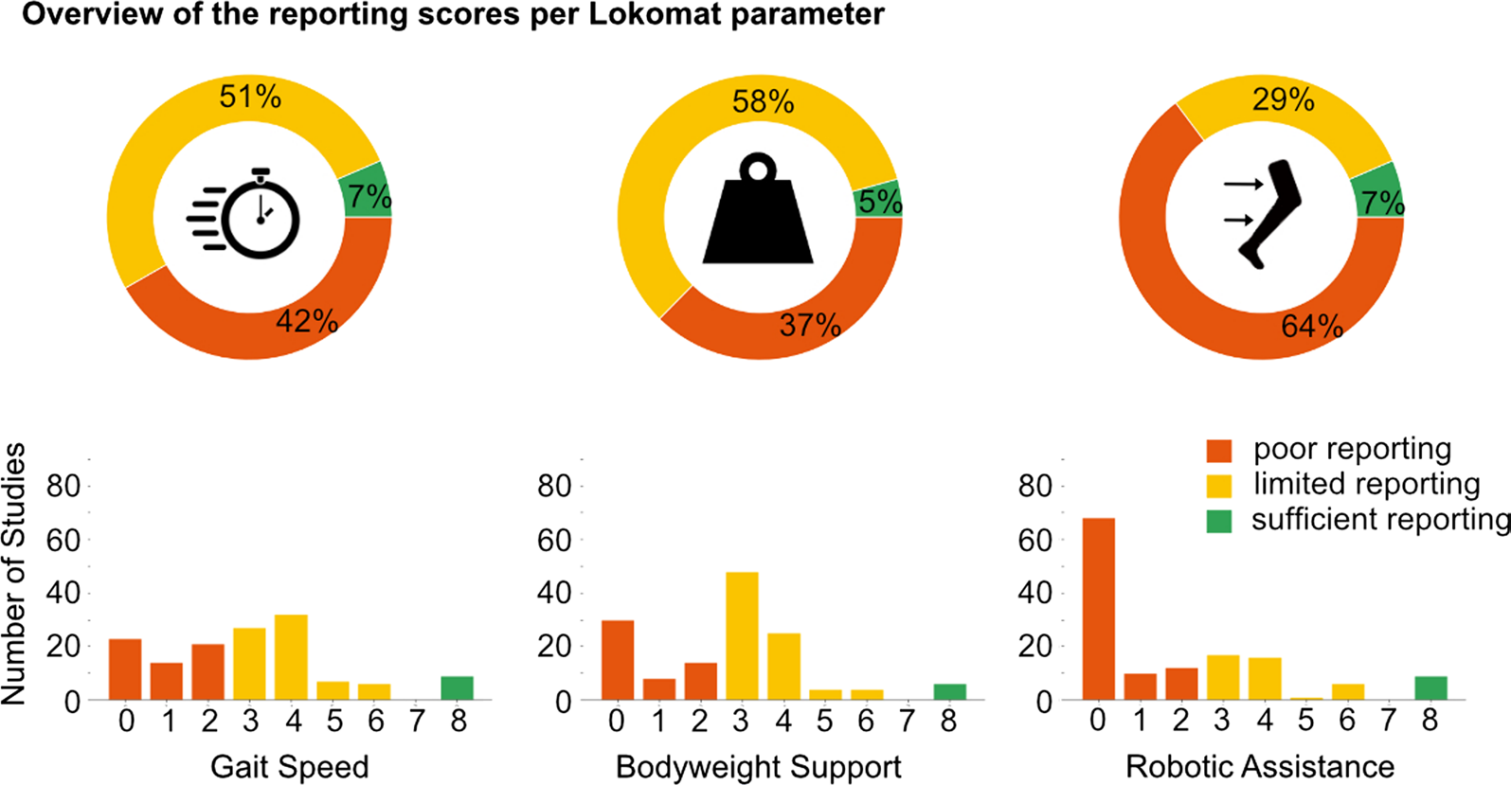
The figure illustrates the distribution of the reporting quality by parameter. All clinical studies were scored for the three categories Gait speed, Bodyweight support and Robotic Assistance with the scheme in Table 1. Scores from 0 to 2 correspond to a poor reporting not eligible to allow a comparison of studies, scores from 3 to 6 to a limited reporting that provide some insights in the therapy approach and allow to transfer the strategy, and scores from 7 to 8 to a sufficient reporting to include the results in a meta-analysis and judge whether the strategy matches the actually performed therapy

A possible alternative approach would be to synthesize results of various studies to evaluate the contributions of therapy parameters on the effectiveness of RAGT. Such a synthesis is currently already being impeded by three main factors: (1) There are many different pathologies involved, (2) many different outcome measures used and (3) different strategies applied as mentioned above. In addition, the finding that only 4–7% of the studies did report parameter settings in a way that they could be used for such a meta-analysis (Fig. 4), makes the clinical transfer of such results very difficult. Even more so, since these 4–7% include mostly studies that applied a fixed parameter setting which clinically rarely makes sense. Even though many studies reported an increase or a decrease in one or more parameters as the therapeutic strategy, a synthesis of the study results without knowing the actual magnitude of such a change would make little sense. For example, a decrease in guidance force from 100 to 80% could have a very different influence on the outcome compared to a decrease from 100 to 30%. We present the available information per study to interested readers in Additional file 3.

Despite the current shortcomings, we believe that meta-analyses including the therapy parameters would have a high potential for further knowledge gain in the future and therefore, the reporting of the therapy parameters in clinical studies should to be improved.

In addition, as illustrated by Fig. 4, depending on the parameter, 39–64% of the studies do not allow any transfer of the therapy strategy employed into the clinical setting. Therapists, who want to know whether or not to use Lokomat therapy for a particular patient, cannot adopt the strategy from studies with promising results, nor can they assess the trustworthiness of the results and compare the choice of the therapy parameters with their own clinical experience. The results of these studies are therefore difficult to translate and as such, their usefulness for therapists is very limited. Despite the poor overall reporting quality on therapy parameters, we would like to encourage RAGT practitioners to take a close look at the therapy content when interpreting clinical study results. Furthermore, an improved reporting would enable an evidence-based translation into Lokomat therapy practice.

The reporting quality assessment framework developed in this review could serve researchers to assess the quality of their reporting before submission of a Lokomat-related paper and could help establishing a minimal information standard similar to the ones of other research areas such as biochemistry [108]. To encourage adoption of such reporting standards by as many scientists as possible, it should be easy to implement and not require much additional effort. We suggest that clinical studies investigating RAGT should at least describe the actual training settings of the three parameters *gait speed*, *body weight support* and *robotic assistance*. Authors should consider reporting at least an average value and a standard deviation for each category per training and subject. This would be equivalent to 7 or more points within the framework presented above. With this fairly simple approach the quality of reporting could be improved. It would allow therapists and researchers to better compare and synthesize studies, as well as facilitate the transfer of promising study findings into clinical practice.

In conclusion, there is an underreporting of therapy parameters in the literature, although there is evidence to suggest that the therapy parameter settings are important. To enable further advances in the field, particularly with regards to the effectiveness of certain parameter settings for individual patient groups, a common minimal reporting standard is proposed. We invite researchers, who are about to publish Lokomat-related research, to make use of the developed reporting framework to check and improve the reporting quality of their work. Furthermore, current evidence suggests that therapy parameters might be important and RAGT practitioners should pay attention to the reported therapy content when translating research findings to their own clinical practice.

## Supplementary Information

**Additional file 1. **PRISMA Checklist for Scoping Reviews.

**Additional file 2. **Search terms.

**Additional file 3. **Information about Settings and Scores per study. Scores from 0 to 2 are marked as red. This group consists of papers that contain a very limited amount of information. In yellow are scores from 3 to 6. These papers report on minimal and/or maximal parameters or the goals that therapists pursue. In green, papers scored with 7–8 are marked. These papers report the actual therapy parameters in a way that they could potentially be used for a meta-analysis.

## Data Availability

Data sharing is not applicable to this article as no datasets were generated or analyzed during the current study.

## Abbreviation

RAGT: Robot-assisted gait therapy
