# Supplementary material for: Settings matter: a scoping review on parameters in robot-assisted gait therapy identifies the importance of reporting standards
Source: J Neuroeng Rehabil. 2022 Apr 22;19:40. doi: 10.1186/s12984-022-01017-3 (PMC9034544; doi:10.1186/s12984-022-01017-3)
Supplement: Supplementary file 2 — Additional file 2. Search terms. [file 12984_2022_1017_MOESM2_ESM.docx]

**Additional File 1:**

**Pubmed:**

( ( patient* ) OR ( "cerebral palsy" [mesh]) OR ( stroke [mesh]) OR ('parkinson disease' [mesh]) OR ('spina bifida' [mesh]) OR (meningomyelocele [mesh]) OR ( "spinal cord injuries" [mesh]) OR ( "brain injury" [mesh]) OR ( "multiple sclerosis" [mesh] ) OR ( disabilit* ) OR ( disorder* ) ) AND ( ( lokomat ) OR ( robot* ) OR ( electromechanical ) ) AND ( ( gait [mesh]) OR ( walking [mesh]) OR (locomotion [mesh]))

**Embase**

('patient'/exp OR 'cerebral palsy'/exp OR 'cerebrovascular accident'/exp OR 'multiple sclerosis'/exp OR 'brain injury'/exp OR 'diseases'/exp OR 'disability'/exp OR 'meningomyelocele'/exp OR 'spina bifida'/exp OR 'parkinson*'/exp) AND (electromechanical OR 'robotics'/exp OR lokomat) AND ('gait'/exp OR 'walking'/exp OR 'locomotion'/exp)

**SCOPUS:**

TITLE-ABS-KEY ( ( ( patient* ) OR ( "cerebral palsy" ) OR ( stroke ) OR ( "spinal cord injur*" ) OR ( "brain injur*" ) OR ( "multiple sclerosis" ) OR ( disabilit* ) OR ( disorder* ) OR (Meningomyelocele) OR ("spina bifida") OR ('parkinson*')) AND ( ( lokomat ) OR ( robot* ) OR ( electromechanical ) ) AND ( ( gait ) OR ( walking ) OR (locomotor)))
